# Material Properties of the Mandibular Trabecular Bone

**DOI:** 10.1155/2014/470539

**Published:** 2014-10-29

**Authors:** Éva Lakatos, Lóránt Magyar, Imre Bojtár

**Affiliations:** ^1^Department of Structural Mechanics, Budapest University of Technology and Economics, Műegyetem Rakpart 3, Budapest 1111, Hungary; ^2^Department of Forensic Medicine, Semmelweis University, Üllői út 93, Budapest 1091, Hungary

## Abstract

The present paper introduces a numerical simulation aided, experimental method for the measurement of Young's modulus of the trabecular substance in the human mandible. Compression tests were performed on fresh cadaveric samples containing trabecular bone covered with cortical layer, thus avoiding the destruction caused by the sterilization, preservation, and storage and the underestimation of the stiffness resulting from the individual failure of the trabeculae cut on the surfaces. The elastic modulus of the spongiosa was determined by the numerical simulation of each compression test using a specimen specific finite element model of each sample. The received mandibular trabecular bone Young's modulus values ranged from 6.9 to 199.5 MPa.

## 1. Introduction

The biomechanical behaviour of a dental implant plays an important role in its functional longevity inside the bone. Implants can have either advantageous or destructive effect on the surrounding bone, depending on several physiological, material, and mechanical factors. The mandible—lower jaw bone—like most human bones is divided into an external cortical and an internal trabecular substance (or spongiosa). The aim of the following experiments was to determine the mechanical properties of the human mandibular trabecular bone, to be used in further finite element models. Application of finite element analysis has become an indispensable method for estimating mechanical behaviour, stress and strain distributions under a certain load, of the cortical and cancellous bone surrounding dental implants, since it is nondestructive. These numerical experiments have their importance in making the implantation the most possibly secure, reliable, and efficient and the lifetime of the implant the longest conceivable, by finding the most favourable thread formation, surface, material, and so forth.

The measurement of the trabecular bone material properties by means of conventional mechanical tests involves several difficulties [1, 2]. Because of the scale of the human bones, the primary difficulty is to obtain cubic shaped specimens from purely trabecular regions larger than 5 mm, which is taken for the lower limit to be examined in compression tests [2, 3]. This can cause serious problems in the examination of the small bones like mandible. In contradiction to the measurements of artificial materials, further difficulties arise in case of biological materials—especially human tissues. Since the poor availability of specimens, the researchers are often under the necessity of drawing conclusions from small number of measurements. The most general laboratorial method for the examination of the bone mechanical properties is the compression test, which contains uncertainties even in the case of ideal shaped samples [1–3]. When purely trabecular samples are harvested, the trabeculae on the edges are cut. Their individual failure causes initial stiffening and the underestimation of Young's modulus [2–4]. In case of several bone types anisotropy and inhomogeneity are hard to estimate previously [1, 2]. Friction between the platform and the specimen leads to uneven stress distribution in the material, which has to be taken into account, when stress-strain pairs are calculated from the force-displacement values; otherwise it leads to the inaccuracy of the obtained Young's modulus values [2–4].

According to the results of previous researches into the measurement of the trabecular bone's elastic properties, a wide range of Young's modulus values can be found (from 1 MPa to 9800 MPa). The experimental values depend on the measuring technique and on several physiologic factors, the results of which are not always clear. These factors are among others: species; anatomical location; the age, sex, and diseases of the donor; the effects of hormones; the density, porosity, and mineral content of the sample; the method of extraction; the preservation and the preparation of the specimen; the measuring technique (strain rates, supports).

The most commonly examined species are cattle [2, 5, 6], sheep [2, 7], swine [2], and canine [2, 8], but results can be found from the examination of monkeys, cats, goats, hares, and rats as well [2]. Most of the samples—either animal or cadaveric human—submitted to compression tests were vertebral [2, 9], femoral [2, 5, 8–11], or tibial [9]. Occasionally can be found measurements concerning the mandible [12–14], ilium [9], or patella [9].

Most of the measurements have been conducted using cubic or cylindrical shaped specimens which have been machined on all faces, thus containing the inaccuracy from the individual failure of the trabeculae cut on the edges. Misch et al. [14] demonstrated the underestimation of the stiffness caused by the machining of the loaded surfaces using cylindrical, mandibular samples (Table 1).

The appropriate sterilization, preservation, and storage of the specimens having the smallest effect possible on the test results are debated. Despite the destructive effect of the ice crystals, the most spread preservation technique is freezing [2, 6, 9, 11, 14]. Embalming by means of various agents [7, 12] and drying [10] are used as well. The removal of the medullary substance slightly affects Young's modulus, when low strain rates are used, while in case of sudden impact liquids in the medullary cavities result in the increase of stiffness [6]. Young's modulus data obtained by the compression tests of fresh samples—without sterilization and preservation—are rare and only available for the vertebral and patellar trabecular bone substances [9].

To determine Young's modulus of the human mandibular trabecular bone, Misch et al. [14] conducted compression tests using cylindrical cadaveric samples. The cylinders were drilled out from the bone in vertical direction and stored frozen. The samples covered by cortical layer resulted in 24.9–240 MPa Young's modulus (mean: 96.2 MPa, standard variation: 40.6 MPa), while the others with machined surface gave 3.5–125.6 MPa (mean: 56.0 MPa, standard variation: 29.6 MPa). O'Mahony et al. [13] conducted compression tests on seven trabecular bone samples (stored frozen) harvested from the mandible of one single cadaver, in three anatomically characteristic directions: inferosuperior (vertical), buccolingual (horizontal, perpendicular to the arch of the mandible), and mesiodistal (horizontal, in the direction of the arch of the mandible). In these three directions Young's modulus values measured were 114, 511, and 907 MPa, respectively. The aforementioned two experiments focused on the arched, implantologically interesting part (corpus mandibulae) of the mandible. On the contrary van Eijden et al. [12] examined trabecular bone samples from the condylar part of the mandible (processus condylaris), which is not involved in implantological treatments but contains trabecular bone in a larger amount. The specimens were preserved by embalming and tested under compression in the horizontal and vertical directions. Young's modulus values reported were 438 MPa and 157 MPa in the horizontal and vertical directions, respectively.

Young's modulus values from the aforementioned experiments are summarized in Table 1.

From the above review it is emerged that no standard measuring technique exists for the mechanical properties of biological tissues as opposed to artificial materials. The aim of the following experiment series was to eliminate the inaccuracies in Young's modulus measurements of the mandibular trabecular bone resulting from the preservation and the machining of the loaded surfaces.

In case of trabecular bone—especially in small bones like mandible—extracting specimens with regular shape and uniform size encounters difficulties. In order to achieve comparable results further examinations might be required.

Besides the loading compression tests, unloading mechanical tests exist, eliminating the plastic effects from the results [28, 29], probably resulting from the sliding between mineral crystals [30] or collagen cross-linking [31]. Ultrasonic measurements might be coupled with mechanical tests or micromechanic models for validation of the test results and for getting closer to the complete, anisotropic elastic properties of the bone or scaffolds [28, 32–34].

In the present research the mechanical behaviour of the human mandible has been examined by means of compression tests. Young's modulus of the trabecular bone substance has been determined from the numerical simulations of the experiments.

## 2. Materials and Methods

**Figure 1:**
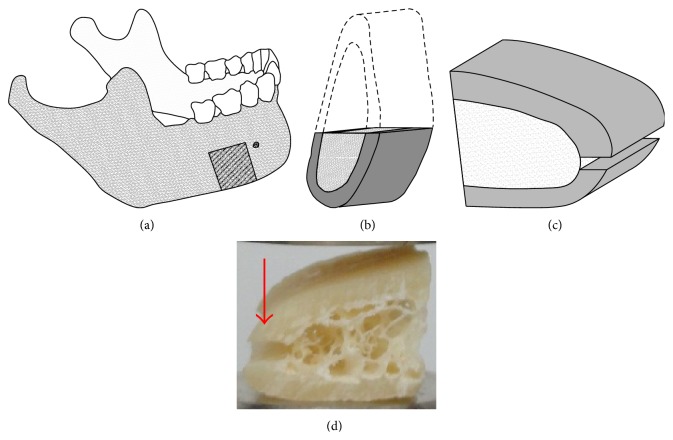
The position of the bone specimens in the mandible (a) and its cross section (b) and the illustration of the cut cortical bone ((c)-(d)).

In the following experiments, fresh cadaveric samples were tested under compression. (Ethics committee approval was obtained—Approval number 4/2011 TUKEB). Ten specimens were harvested from the molar mandibular region of 6 middle aged male patients from the lower edge of the bone. Since we aimed to examine the trabecular bone, the cortical layer around it was cut, the way it is shown in Figure 1.

**Figure 2:**
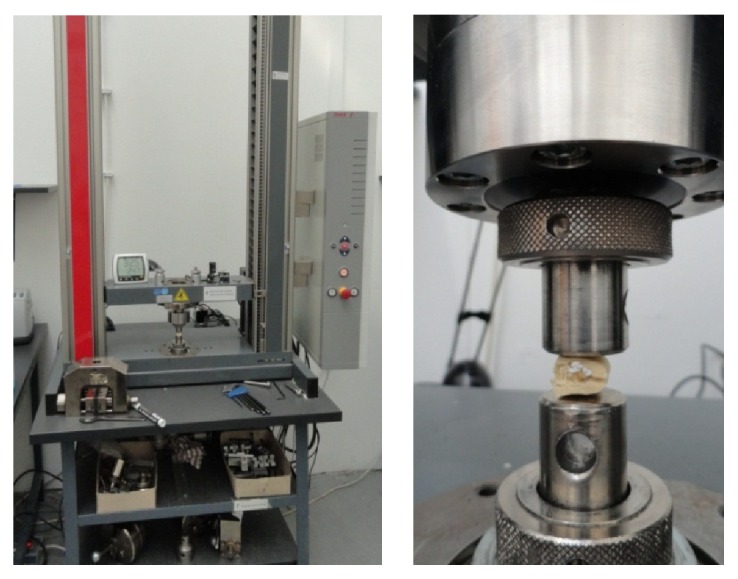
The testing machine and an illustration of the compression test (for better visibility demonstrated on a dried sample).

**Figure 3:**
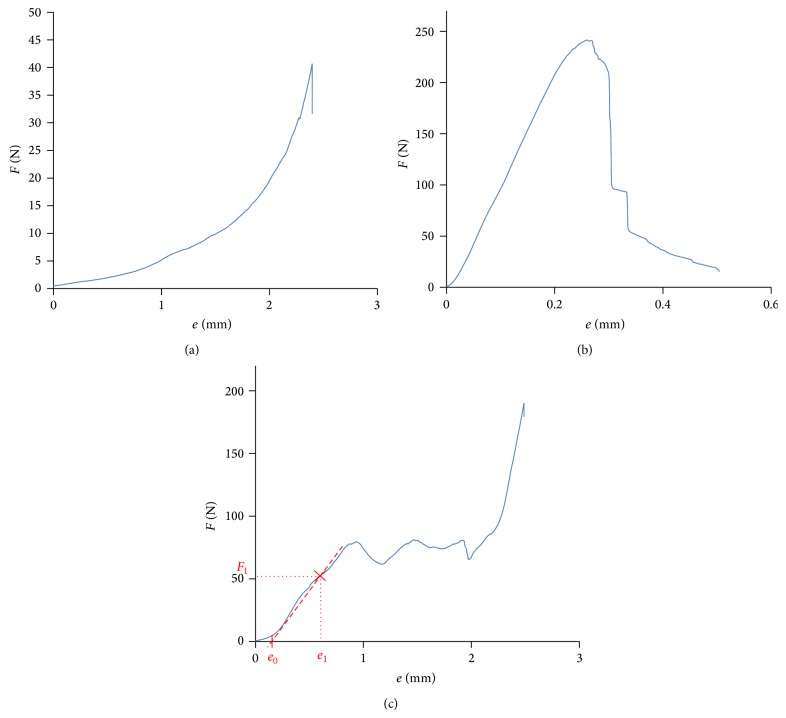
Force-displacement diagrams detected: too soft (a) and too stiff (b) trabecular bone and the results of the successful tests corresponding the diagram of compressed cellular solids (c).

The samples were submitted to compression tests using a Zwick Z005 displacement controlled testing machine (Figure 2), and force-displacement pairs were registered using 0.5 mm/min loading rate. The tests resulted in three basic types of force-displacement curves, two of which (Figures 3(a) and 3(b)) were the compression of samples with poor or no trabecular substance (Figure 3(a)) and with so stiff cancellous bone, which rather possesses the characteristics of compressed cortical bone (Figure 3(b)). Three measurements like these were excluded from the further examinations.  Figure 3(c) shows a typical example of the received force-displacement curves from the successful measurements, which corresponds to the characteristic diagram of the compressed cellular solids [28]. The initial, closely linearly elastic part comes from the elastic bending of the trabeculae and the long horizontal plate shows the gradual failure of the spongiosa, until the cell walls touch and the curve increases steeply.

## 3. Results

**Figure 4:**
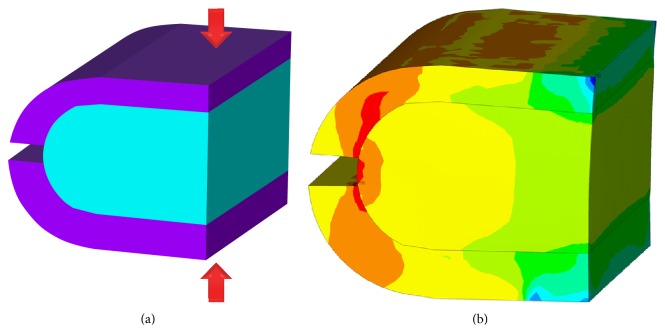
Specimen specific finite element model for Young's modulus calculations (a) and the vertical normal stress distribution from the vertical compressive load—the highest compressive and tensile stresses are indicated in dark blue and red through green, yellow, and orange (b).

Since the geometry of the specimens was complex and varying, the numerical simulation—using the ANSYS software system—of each compression test was conducted to determine Young's modulus of the trabecular bone. For the simulations a parametric finite element model (Figure 4) was created, which possesses variable geometrical properties, set according to the original bone sample. The dimensions measured on the specimens were as follows: outer extents of the sample, the bearing length of the load, and the cortical thickness on the buccal and lingual sides and by the cut. Both the cortical and the trabecular bone materials were assumed to be linearly elastic continuums with Poisson's ratio 0.3 [2]. The elastic properties of the cortical layer were set according to data from literature: a 15 GPa Young's modulus value was used [29].

Young's modulus of the trabecular bone was determined by simulating the compression test (Figure 4): loading the upper side of the model with vertical force and constraining the lower side against horizontal and vertical displacements. An arbitrary force (*F*_1_) value from the initial elastic part of the *F*-*e* diagram (Figure 3(c)) was applied and Young's modulus of the spongiosa was set to result in the same displacement (*e*_1_ − *e*_0_) as the compression test did (where *e*_0_ is a displacement from the initial balancing, resulting from the inaccuracy of the specimen geometry). The elastic modulus was found using the following iteration algorithm, initiating from an arbitrary *E*_1_ value:

(1) $$\begin{array}{c} E_{i+1}=\frac{E_{i}}{\left(e_{1}-e_{0}\right)/U_{zi}},\;\text{while}\;\;\left|\Delta e\right|>ɛ, \end{array}$$

where *E*_*i*_ is Young's modulus of the trabecular bone in the *i*th iteration step, (*e*_1_ − *e*_0_) is the displacement of the real bone from the experiments, introduced above, *e*_0_ is a displacement from the initial balancing, *U*_*zi*_ is the displacement of the top point of the structure in the *i*th iteration step, |Δ*e*| = (*e*_1_ − *e*_0_) − *U*_*zi*_ is displacement error, and *ɛ* is a predefined accuracy.

The received mandibular trabecular bone Young's modulus values ranged from 6.9 to 199.5 MPa (namely, 20.29, 199.5, 61.4, 26.7, 6.9, 49.7, and 8.5 MPa). Compared to the literature of the cancellous bone mechanical properties, the results show correlation to the values—24.9 to 240.0—measured by Misch et al. (1999) [14, 30]. The method introduced above provides results in the buccolingual direction. The elastic modulus values were determined to be used in further finite element simulations.

## 4. Discussion

The present research aimed at overcoming some difficulties of determining the trabecular bone material properties by means of conventional mechanical tests and to give a closer estimation of Young's modulus of the trabecular substance of the human mandible, narrowing the wide range of values that can be found in the literature. The specimens were protected from the destruction caused by sterilization, preservation, and storage, by using no freezing or embalming on them. The compression tests were conducted on the fresh cadaveric samples immediately after extraction. To avoid the underestimation of Young's modulus caused by the individual failure of the trabeculae cut on the surfaces, the samples were covered with cortical layer.

Young's modulus of the trabecular bone inside the complex and varying shaped specimens was determined by the numerical simulation of each compression test using the specimen specific finite element model of each sample with geometrical properties set according to the original bone sample. An iteration algorithm—initiated from an arbitrary elastic modulus value—was followed, until the simulated compression test resulted in the same displacement from the same load as the experiment. The simulations resulted in Young's modulus values comparable to the measurements of Misch et al. (1999) [14, 30].

## Figures and Tables

**Table 1:** Experimental Young's modulus values [2, 5, 6, 9, 10, 12–14].

Author	Bone type	Preservation	Young's modulus
Evans and King, 1961 [15]	Femur	Embalmed	20.68–965 MPa
McElhaney et al., 1970 [16]	Vertebra	Fresh	Avg 151.7 MPa
Pugh et al., 1973 [17]	Femur	Frozen	423–1516 MPa
Schoenfeld et al., 1974 [18]	Femur	Fresh	Avg 344,7 MPa
Lindahl, 1976 [19]	Tibia	Dried, defatted	1.4–79 MPa
Lindahl, 1976 [19]	Vertebra	Dried, defatted	1.1–139 MPa
Carter and Hayes, 1977 [6]	Tibia	Frozen	10–500 MPa
Ducheyne et al., 1977 [20]	Femur	Frozen	58.8–2942 MPa
Brown and Ferguson, 1980 [21]	Femur	Frozen	1000–9800 MPa
Williams and Lewis, 1982 [22]	Tibia	Dried, defatted	8–457 MPa
Goldstein, 1987 [9]	Tibia	Frozen	4–430 MPa
Martens et al., 1983 [23]	Femur	Frozen	58–2248 MPa (900 ± 710 MPa)
Ciarelli et al., 1986 [24]	Tibia	Frozen	5–552 MPa
Ciarelli et al., 1986 [24]	Femur	Frozen	7.6–800 MPa
Ciarelli et al., 1986 [24]	Radius	Frozen	1.1–448 MPa
Ashman and Rho, 1988 [5]	Vertebra	Fresh	158–378 MPa
Keller et al., 1987 [25]	Vertebra	Frozen	15–30 MPa
Struhl et al., 1987 [26]	Vertebra	Frozen	10–428 MPa
Odgaard and Linde, 1991 [4]	Femur		103–1058 MPa
Linde, 1994 [3]	Tibia		445 ± 256 MPa
Keaveny et al., 1997 [27]	Vertebra		165 ± 110 MPa
Misch et al., 1999 [14]	Mandible	Frozen	24.9–240 MPa (with cortical layer) 3.5–125.6 MPa (without cortical layer)
O'Mahony et al., 2000 [13]	Mandible	Frozen	Avg 907 MPa (mesiodistal) Avg 511 MPa (buccolingual) Avg 114 MPa (inferosuperior)
van Eijden et al., 2004 [12]	Mandibular condyle	Embalmed	Avg 438 MPa (vertically) Avg 157 MPa (horizontally)
Chevalier et al., 2007 [10]	Femur	Dried, defatted	63.9–2987.9 MPa
